# Sustainability Assessment of the End-of-Life Technologies for Biocomposite Waste in the Aviation Industry

**DOI:** 10.3390/polym15122689

**Published:** 2023-06-15

**Authors:** Špela Ferjan, Milkica Jovičić, Nora Lardiés Miazza, Tom Ligthart, Clare Harvey, Sergio Fita, Rajesh Mehta, Pouya Samani

**Affiliations:** 1 Energy and Materials Transition (EMT), Netherlands Organisation for Applied Scientific Research (TNO), 3584 CB Utrecht, The Netherlands; spela.ferjan@tno.nl (Š.F.); tom.ligthart@tno.nl (T.L.); clare.harvey@tno.nl (C.H.); rajeshmehta@sustainovationventures.com (R.M.); 2 Sustainable Processes and Energy Systems (SPES), Netherlands Organisation for Applied Scientific Research (TNO), 2628 CA Delft, The Netherlands; milkica.jovicic@tno.nl; 3 AIMPLAS Asociación de Investigación de Materiales Plásticos y Conexas, València Parc Tecnològic, 46980 Paterna, Spain; nlardies@aimplas.es (N.L.M.); sfita@aimplas.es (S.F.)

**Keywords:** basalt, bioepoxy, solvolysis, pyrolysis, LCA, TEA

## Abstract

Biocomposites have emerged as promising alternative materials for the aviation industry. However, there is a limited body of scientific literature addressing the end-of-life management of biocomposites. This article evaluated different end-of-life technologies for biocomposite recycling in a structured, five-step approach applying the innovation funnel principle. First, ten end-of-life (EoL) technologies were compared in terms of their circularity potential and technology readiness levels (TRL). Second, a multi-criteria decision analysis (MCDA) was carried out to find out the top four most promising technologies. Afterwards, experimental tests were conducted at a laboratory scale to evaluate the top three technologies for recycling biocomposites by analysing (1) three types of fibres (basalt, flax, carbon) and (2) two types of resins (bioepoxy and Polyfurfuryl Alcohol (PFA) resins). Subsequently, further experimental tests were performed to identify the top two recycling technologies for the EoL treatment of biocomposite waste from the aviation industry. Finally, the sustainability and economic performance of the top two identified EoL recycling technologies were evaluated through life cycle assessment (LCA) and techno-economic analysis (TEA). The experimental results, performed via the LCA and TEA assessments, demonstrated that both solvolysis and pyrolysis are technically, economically, and environmentally viable options for the EoL treatment of biocomposite waste from the aviation industry.

## 1. Introduction

If the aviation industry was a country, it would be among the top ten carbon-polluting countries in the world [1]. The lightweight design of aircraft is a strong lever to lower fuel consumption and its associated environmental impacts. Therefore, FRP (fibre-reinforced polymer) composites have been widely used in these sectors, as they can provide the required mechanical properties while having a low weight. Despite the favourable product properties, the end of life of FRP composites is often limited to downcycling (e.g., using mechanically shredded FRP as fillers) or recovering the carbon fibres (due to their higher market price and demand). This can be explained by the fact that the polymer matrices are usually thermosetting resins with a cross-linked molecular structure, which poses a challenge to conventional recycling technologies. According to the International Air Transport Association (IATA), approximately 700 aircraft reach the end of their operational lives every year. This number is predicted to increase to 15,000 by 2034. A crisis such as COVID-19 would intensify the current end-of-life challenges with composite waste in the aviation industry [2,3]. Further, waste management and evaluation in the aviation industry has mainly focused on pre-consumer waste during manufacturing. However, less attention has been paid to the post-consumer waste at the end-of-life phase of aeroplanes, thereby limiting the opportunities to increase the circularity performance of the composites and biocomposites used in the industry.

Biocomposites have emerged as promising materials compared to FRP composites due to their lower environmental footprint and relatively higher mechanical performance [4,5,6]. Moreover, conventional composites are usually difficult to recycle, and strict environmental legislations and consumer pressure have triggered a paradigm shift toward using more sustainable composites such as biocomposites [7,8]. Biocomposites must have at least one of the components, i.e., the matrix or fibre, to be biobased [9]. Some examples of such components are bio-resins and natural fibres such as flax, hemp, and kenaf. Biofibres have attracted lots of attention due to their abundance, moderate cost, and high strength-to-weight ratio [10,11,12,13]. Despite the potential environmental benefits of biocomposites, their end-of-life paths have been less investigated. It should be noted that in addition to the challenges associated with conventional FRP composites due to characteristics such as heterogeneity, contamination, and thermosetting nature, biocomposites do not benefit from economically beneficial recycling options, such as the recovery of carbon fibre material. Therefore, more research is needed in investigating the feasibility of different end-of-life technologies for biocomposite waste. This article aimed to evaluate the different end-of-life paths of biocomposites in the aviation industry. Subsequently, the research question was defined as “Which end-of-life option is the most sustainable for the biocomposite waste from the aviation industry?”.

## 2. Methods

**Figure 1:**
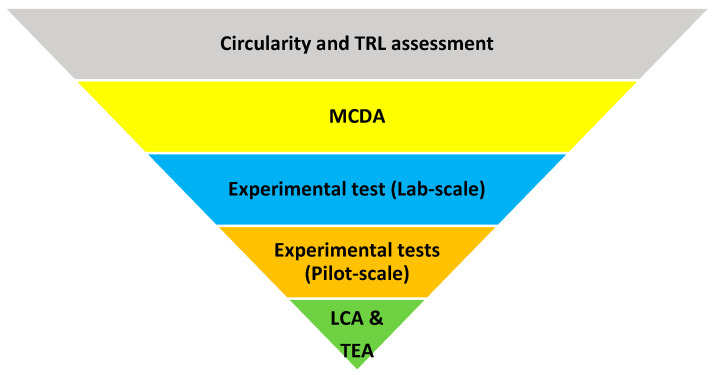
Research design methodology for biocomposite recycling.

The methodology for evaluating different end-of-life paths for biocomposites is shown in Figure 1. Firstly, the circularity and technology readiness levels (TRL) were evaluated for ten different technologies. Then, a multi-criteria decision analysis (MCDA) was carried out to find out the top four most promising technologies. Afterwards, experimental tests were conducted at a laboratory scale to evaluate these technologies. Subsequently, additional experimental tests were conducted to identify the final candidates for the EoL treatment of biocomposite waste (two alternatives). Finally, the sustainability of the identified alternatives was evaluated through life cycle assessment (LCA) and techno-economic analysis (TEA). The methodological steps are explained in the following sections.

### 2.1. Circularity and TRL Assessments

To assess different end-of-life paths for biocomposite recycling, different end-of-life options with the transferability potential for the treatment of FRP biocomposites were first reviewed and compared. This included evaluating their TRL and circularity potential. The evaluated end-of-life paths comprised mechanical recycling, combustion in a cement kiln, three types of pyrolysis (conventional, fluidised bed, and microwave), gasification, enzymatic degradation, composting, solvolysis, and dissolution.

### 2.2. Multi-Criteria Decision Analysis (MCDA)

After evaluating the circularity and TRLs of alternative end-of-life paths, numerous MCDA criteria were selected through stakeholder elicitation, the development of a questionnaire consisting of a list of criteria, and by asking technology experts to select the most influential criteria and their associated weights. These criteria were categorised into four groups of technical, economic, resource, and environmental aspects and were evaluated in either quantitative form or qualitative form based on a Likert scale (e.g., very high to very low). All these scales were then transferred to a five-point scale, in which the highest value represents the best performance. Table 1 shows the defined criteria and their allocated weights for the MCDA. The selection of these criteria and weighting factors were given by an expert panel. The MCDA technique has been used for identifying the best alternatives concerning various criteria in different areas, including the composite sector [14].

Afterwards, an MCDA was conducted by comparing the alternatives for the treatment of 1 ton of FRP biocomposite waste and taking into account the collection of the disposed FRP composite, as well as the transport, sorting, cleaning, pre-treatment processes, and ultimately the treatment of the final waste. After screening the alternative materials, bioepoxy was selected as the matrix and basalt twill weave and flax tape as two types of fibres as the reference materials to provide a comparison. Then, the overall feasibility level *OFL_j_* for each alternative (end-of-life path) was calculated by the following Equation (1):

(1) $$OFL_{j}=\underset{i}{\sum }W_{i}S_{ij}$$

where *W_i_* represents the allocated weight for each criterion, and *S_ij_* is the score for method *j* according to category *i*. The allocated weights by the expert panel for different aspects are presented in Table 2.

### 2.3. Experimental Testing

After theoretical assessments, as explained in the previous sections, the screening of alternative experimental tests was designed to evaluate the technical feasibility of the selected four technologies, namely pyrolysis, solvolysis, dissolution, and mechanical recycling. The experimental tests were conducted first on the laboratory scale and then on the pilot scale.

#### 2.3.1. Laboratory Scale Testing

The selected EoL technologies by the MCDA were evaluated for the following three different biocomposites: (1) flax fibre with a bioepoxy matrix, (2) basalt fibre with a PFA matrix, (3) and carbon fibre with a bioepoxy matrix. The testing trials allowed us to evaluate different processing parameters and to optimise them for each EoL method.

**Figure 2:**
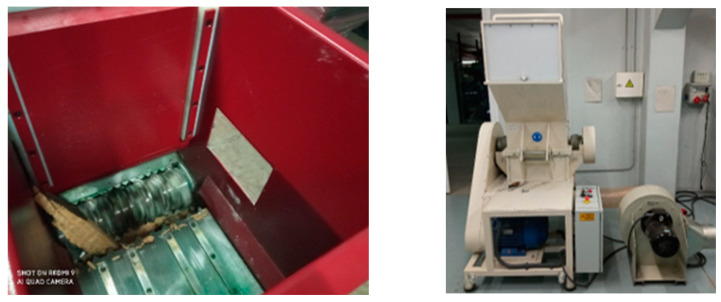
Shredding (**left**) and milling (**right**) equipment in AIMPLAS facilities.

**Figure 3:**
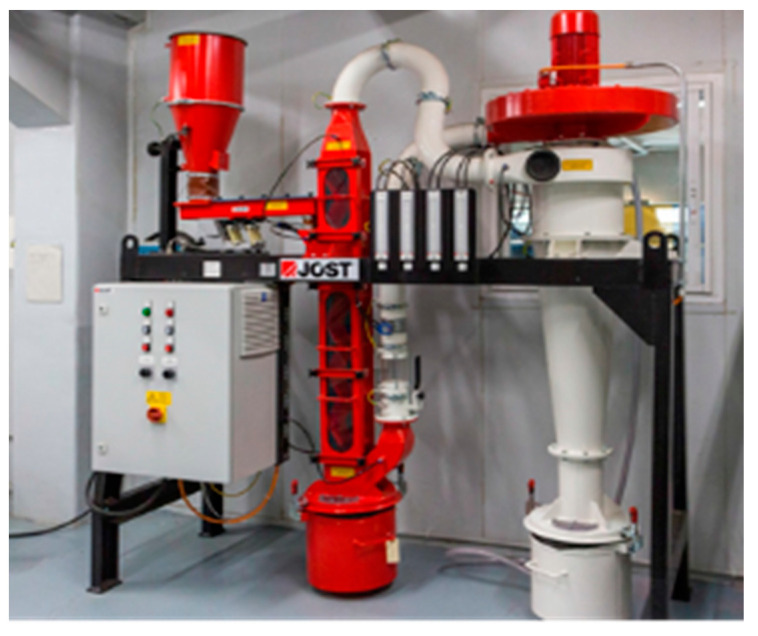
ZigZag equipment for sorting in AIMPLAS facilities.

For evaluating mechanical recycling, shredding and milling (shown in Figure 2) were first conducted to cut down the selected biocomposites up to 5 mm. Afterwards, density-based Zigzag (shown in Figure 3) equipment was used for sorting and dividing the ground biocomposites into fine and coarse particles. Then, the use of these ground biocomposites as filler was evaluated.

**Figure 4:**
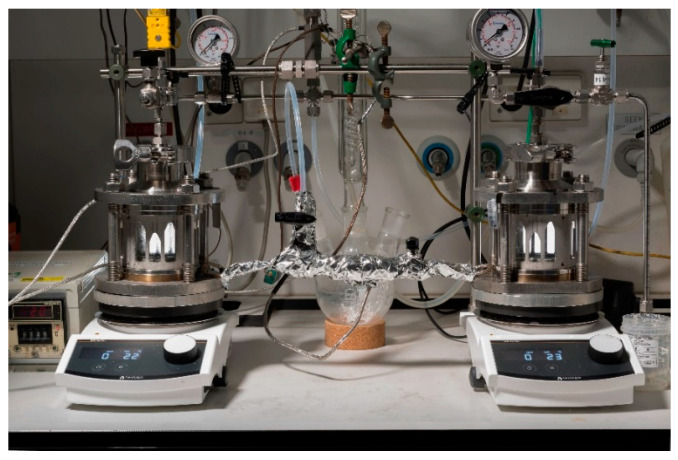
TNO Möbius technology for evaluating the dissolution of the biocomposites.

For assessing the dissolution technology for physical recycling, different solvents were evaluated for two types of matrices, i.e., bioepoxy and PFA. The list of evaluated solvents is provided in Table 3. Then, the selected solvents were examined on biocomposites using TNO Möbius technology, a solvent-based physical recycling technology at the lab scale (shown in Figure 4). For sorted and cleaned plastic waste, the low-viscosity solution in this technology enables filtration and sorption to remove additives and impurities. Afterwards, the polymer precipitates by flash evaporation and the solvent is recovered. In this experiment, firstly a pressurised test tube was filled with 5 wt% of the investigated material and 3 mL of the tested solvent. Then, the composition was heated while stirring. Utilising a pressurised system enabled the solvent to be heated above its boiling point while dissolving the biocomposite, resulting in a more efficient dissolution process.

**Figure 5:**
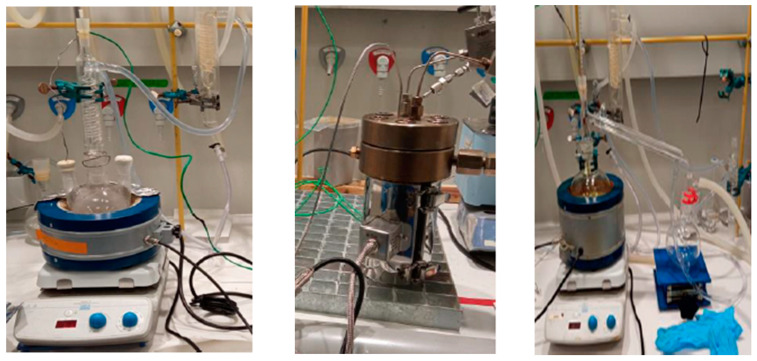
Solvolysis at the laboratory scale: backflow (**left**), reactor (**centre**), distillation system (**right**).

The solvolysis testing was conducted by pre-treatment (i.e., grinding and sorting) of the biocomposites first and before performing the reaction in a reactor. Two conditions of (1) the mixture of Acetic acid and hydrogen peroxide as the solvent and 65 °C as the temperature, and (2) the mixture of acetone and hydrogen peroxide as the solvent and 100 °C as the temperature were examined, as is shown in Figure 5. Then, the samples went through filtration and distillation to eliminate the nonreactive substances and to remove the solvent. Finally, the characterisations were conducted by TGA (Scanning Electron Microscope) and SEM (Scanning Electron Microscope); for the fibre and elemental analyses, Fourier Transform Infrared (FTIR) spectroscopy and Gas chromatography-mass spectrometry were used.

**Figure 6:**
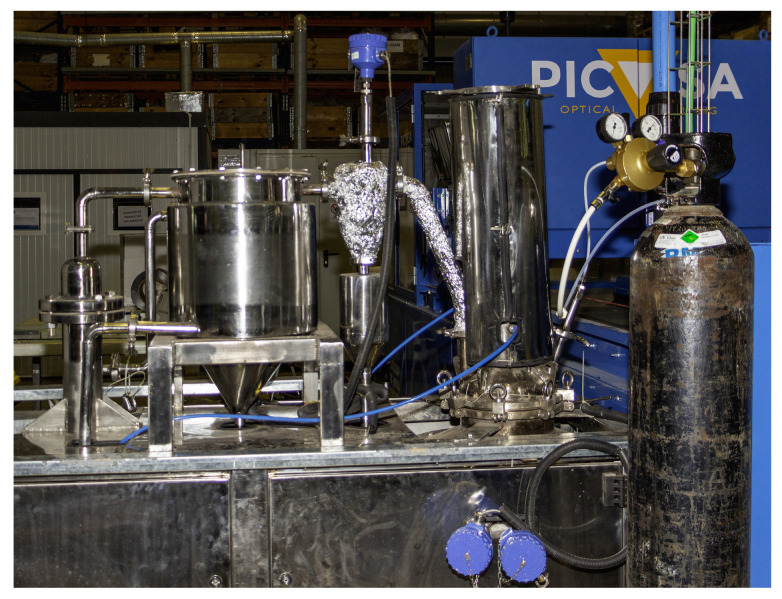
Pyrolysis at the laboratory scale in AIMPLAS facilities.

For evaluating pyrolysis, first, pre-treatment (i.e., grinding and sorting) of the biocomposites was conducted similarly to solvolysis. Then, the absence of contaminants, such as halogenated components, was analysed by characterising the residue by elemental analysis. Afterwards, pyrolysis was carried out in the reactor (as shown in Figure 6). The same tests, as used for the solvolysis process, were conducted to analyse the liquid and solid fractions and determine the composition of the pyrolytic oil and the presence of remaining resin or char over the fibres.

#### 2.3.2. Pilot Scale Testing

**Figure 7:**
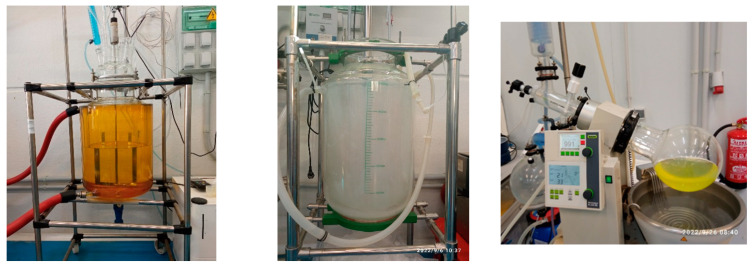
Solvolysis at the pilot scale: 25 L glass reactor (**left**), 50 L glass reactor (**centre**), and rotary evaporator (**right**) in AIMPLAS facilities.

After the laboratory scale testing, EoL technologies, which demonstrated suitability for the evaluated biocomposites, were selected for evaluation at the pilot scale. Pyrolysis and solvolysis were identified as two candidates for this assessment. Moreover, basalt fibre with a PFA matrix and carbon fibre with a bioepoxy matrix were selected as two final alternatives for the biocomposite. The basalt/PFA biocomposite consisted of 10% resin weight (PFA) and 90% fibre weight (basalt). On the other hand, the carbon/bioepoxy biocomposite consisted of 38% resin weight (bioepoxy) and 62% fibre weight (carbon). Similar to the laboratory scale testing, these biocomposites were ground before the reactions. The steps of both processes were similar to the ones at the laboratory scale. Correspondingly, the same characterisation tests and solvent were used for both processes. For solvolysis, two glass reactors, as well as a distillation system (rotary evaporator), were used, as shown in Figure 7. Moreover, similar solvents to the laboratory scale were considered.

### 2.4. Sustainability Assessment

**Figure 8:**
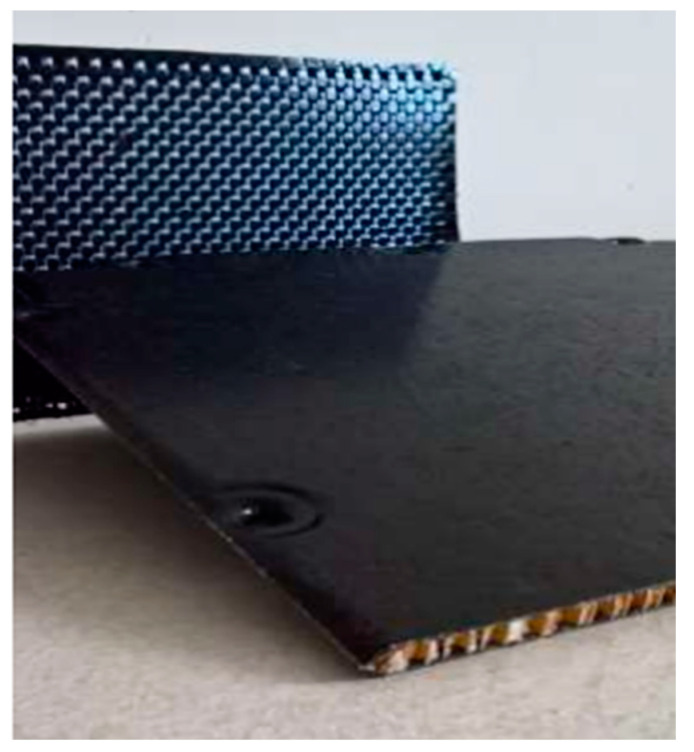
Biocomposite made of basalt fibre and bio PFA resin.

After the identification of the final alternatives at the pilot scale, a sustainability assessment was carried out to obtain insights into the environmental (through LCA) and economic (through TEA) performances of these technologies. Noting the outcomes of the previous sections, pyrolysis and solvolysis were selected as the EoL technologies. Additionally, a biocomposite consisting of basalt PFA biocomposite was considered as the final alternative. This biocomposite (shown in Figure 8) consists of a basalt twill weave, with a 90% weight percentage, and bio PFA resin as the matrix, manufactured by vacuum bagging. A scaled-up demo plant level (TRL 7) with a 10kta feed was the basis for this sustainability assessment.

**Figure 9:**
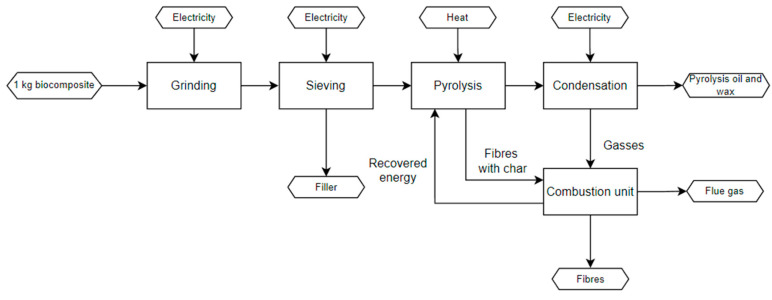
Process flow diagram of the pyrolysis technology for recycling biocomposites.

Figure 9 depicts the steps of the pyrolysis process. A pre-treatment step included grinding the biocomposite to 20 mm in a milling machine and then sieving to further separate bigger parts from the smaller ones. Additionally the material loss during the grinding process was collected to be used as polymer filler. The temperature of the pyrolysis reactor was set to 550 °C in anoxic conditions for a duration of 1 h. The products of this process were pyrolysis oil and wax, as well as a solid residue. The solid residue consisted of char and basalt fibres. Fibres with char and non-condensable gases were then combusted to remove the char from the fibres through oxidation and providing extra heat to be used for the process (energy recovery).

**Figure 10:**
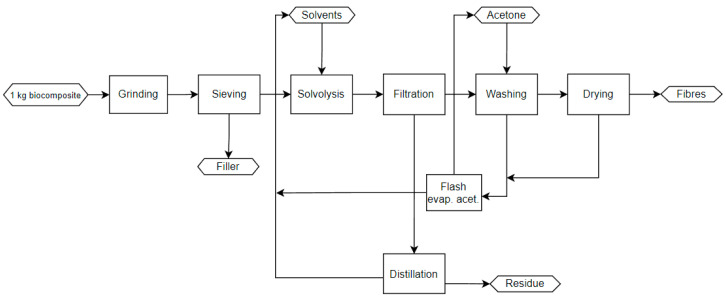
Process flow of the solvolysis technology for the recycling of biocomposites.

Figure 10 shows the steps of the solvolysis process. Similar to pyrolysis and based on the optimised conditions, the biocomposites were ground to 15 mm in a milling machine and then sieved to further separate the bigger parts from the smaller ones. Moreover, the material loss during the grinding process was collected to be used as polymer filler. The selected solvent was a mixture of hydrogen peroxide, water, and glacial acetic acid, and the temperature of the solvolysis reactor was set to 65 °C for the duration of 38 h. Subsequently, the products were filtrated to separate the fibres from the liquids. As the fibres absorbed the solvent mixture, acetone washing was conducted. By further distillation and separation through a flash evaporation unit, acetone and solvents were circulated back to the washing step and solvolysis reactor, respectively, and the lost fractions were sent to wastewater treatment. The fibres were finally dried, and the residue was collected to be used as wax in asphalt.

#### 2.4.1. Life Cycle Assessment (LCA)

The LCA study was conducted in accordance with ISO 14040 and 14044 standards [15,16] to evaluate the environmental performance of pyrolysis and solvolysis. The functional unit was set to “treatment of 1 kg of biocomposite waste”, and the reference flows were equal for both technologies. The system boundaries were set to the EoL phase. Upstream processes, such as the removal of the composite from the aeroplane and waste collection and separation, were excluded from the LCA study, as they are equal for both alternatives, and the ultimate goal of LCA is to compare the recycling technologies. Primary data were used for the foreground processes, and the ecoinvent 3.8 database was used for secondary data. The LCA study was modelled by using SimaPro software version 9.03.03, and the avoided burden approach (system expansion) was utilised for allocation. Global warming potential (GWP) was considered the main impact category for the life cycle impact assessment (LCIA) in accordance with the characterisation factors from the IPCC (Inter-governmental Panel on Climate Change). The inventory data for pyrolysis and solvolysis can be found in Table A1 and Table A2, respectively.

#### 2.4.2. Techno-Economic Analysis (TEA)

The TEA study was based on the defined industrial processes for pyrolysis (Figure 9) and solvolysis (Figure 10) for a fully scaled-up process implemented in a 10 kta plant, and it covered a number of operations that allowed the transformation of the feedstock to the final product(s). Technical analysis described which process steps, process equipment, and (raw) materials are required for recycling the biocomposite, consisting of 90 wt.% basalt fibre and 10 wt.% PFA matrix. The technical analysis covered the storage of raw materials, mechanical recycling (grinding, sieving), and the pyrolysis/solvolysis process. In the case of solvolysis, solvent recovery, storage of final products, and auxiliary equipment were considered as well.

Economic analysis provides information to decision-makers on the requirements for building an industrial plant and its associated costs. For this purpose, the economic life of the plant was set to 10 years, and the plant availability was set to 91%. The cost of recycling biocomposite was set to EUR 0/kg, as a conservative estimation given that it is not clear if the feedstock would need to be bought or the recycler would be paid for waste processing. The price of recovered fibre product was assumed to be EUR 6/kg, where the current market price is in a range between EUR 4 and 11/kg. The price of the recovered filler was given a value of EUR 1/kg, which is on the higher side of the range.

## 3. Results and Discussion

This section summarises the results of the circularity and TRL assessments of the ten studied end-of-life methods for composites and biocomposites. Subsequently, the results of MCDA are presented. Then, the results of the experimental tests are presented. Finally, the results of the LCA and TEA are discussed.

### 3.1. Circularity and TRL Assessments

#### 3.1.1. Mechanical Recycling

Mechanical recycling requires low cost and energy in comparison with alternatives, such as chemical recycling or energy recovery units. It is a well-established technology and has been used in FRPs with TRLs of 8 for carbon fibre and 7 for glass fibre. High speed and the capacity for large volumes are its other advantages. However, the quality of the resulting product depends on various factors such as the prior separation and the presence of impurities. Therefore, the use of recycled product for high-end applications may be limited. The mechanical properties of the fibre rest on their length and are affected by its reduction. Depending on the size of the fibres, the recycled material can be used in different applications as filler or reinforcement (downcycling). This includes filling and reinforcements for concrete and road asphalt (CFRP), tiles and bricks (CFRP and GFRP), thermosetting compounds such as unsaturated polyester panels (GFRP), thermoplastics such as functional filling of PMC (Phenolic Moulding Compound), and the substitution of calcium carbonate for recycled material (GFRP).

#### 3.1.2. Combustion in Cement Kiln

When it comes to FRPs and cement kilns, glass fibre is considered a suitable alternative to fossil fuels due to its high calorific value, low cost, and wide availability. On the other hand, carbon fibres are commonly used for higher-value products because of their price and different chemical composition. Noting the energy intensity of cement kiln processes, using biowaste materials provides novel opportunities for reducing GHG emissions. This is particularly significant because of the high TRL (8–9) and established know-how and infrastructure for GFRP, which can be used for biocomposites as well. On the other hand, this solution is associated with combustion and emissions and, therefore, is not a circular approach. Moreover, the raw material recovery potential is yet limited, and biofibres can be used mostly as a renewable fuel in the cement kiln. Basalt fibre can be used as a feedstock as well.

#### 3.1.3. Classic Pyrolysis

Classic pyrolysis is the most widespread thermal recycling method, and unlike combustion, it occurs in the absence of oxygen. The use of pyrolysis for the end-of-life treatment of CFRP and GFRP composites is well-established in the industry with the TRLs of 7 for glass fibre and 8 for carbon fibre. There is no need for chemical solvents, which is beneficial in lowering the operational risks and environmental impacts. Pyrolysis also can deal with mixed and contaminated waste and provides possibilities to recover both fibre and resin. The converted resin can be used as fuel or even sometimes chemical feedstock. However, refining and purifying these products is challenging and can only be cost-effective on a large scale. The surface and mechanical properties of the retrieved fibres are also degraded. The lower thermal stability of natural fibres, in comparison with glass and carbon fibres, needs to be tackled for using this technology for the end-of-life treatment of biocomposites. The TRL of this technology is estimated to be 3 for biocomposites. Noting the long duration required for pyrolysis, the emergence of varieties such as fluidised-bed and microwave-assisted techniques provides new opportunities for its further application in different sectors, such as the aviation industry and treatment of biocomposite waste.

#### 3.1.4. Fluidised-Bed Pyrolysis

Fluidised-bed pyrolysis provides a higher efficiency of the heating process in comparison with classic pyrolysis. This allows the floating of the waste material at a lower temperature and better control of the system. Moreover, this technology can be used for mixed and contaminated FRP composite waste, with painted surfaces or foam cores in sandwich-structured composites. Fluidised-bed pyrolysis can be used for both CFRP and GFRP composites. However, the fibres are more damaged due to the temperature and attrition of the fluidised sand and, consequently, have poorer mechanical properties. Additionally, recovering products from the resin, as classic pyrolysis allows, is not possible. Noting the lower thermal degradation of natural fibres, in comparison with carbon and glass fibres, fluidised-bed pyrolysis is not an ideal end-of-life treatment process for biocomposite waste. The TRL of this technology is estimated to be 4 for FRP composites and 3 for biocomposites.

#### 3.1.5. Microwave-Assisted Pyrolysis

Microwave-assisted pyrolysis has emerged as a promising variant of classic pyrolysis that provides faster thermal transfer and chemical reaction and, consequently, lower energy requirement. This technology has proven to be efficient in recycling both CFRP and GFRP waste. The recycled carbon fibre has clear fibre surfaces and mechanical properties comparable with virgin fibres. The recycled glass fibre also shows improved mechanical properties in comparison with classic pyrolysis. Moreover, the chemical feedstock can be retrieved from the resin. While this technology is suitable for mixed and contaminated FRP composite waste, the reaction of different polymer coatings to microwave heating still needs to be investigated. Moreover, similar to fluidised-bed pyrolysis, the lower thermal degradation of natural fibres is challenging in using this technology for biocomposite waste. The TRL of this technology is estimated to be 3 for FRP composites and 2 for biocomposites.

#### 3.1.6. Gasification

Gasification has been applied to different types of composites to separate fossil-based resins from carbon and glass fibres. The mechanical properties of the fibres are, however, affected due to the high temperature and the presence of char or resin residues over the fibre. In comparison with pyrolysis, gasification leads to a higher gaseous fraction and a lower oil fraction. Similar to advanced pyrolysis technologies, using high temperatures makes the use of gasification for biofibres lead to thermal degradation. The TRL of this technology is estimated to be 8 for FRP composites and 4 or 5 for biocomposites.

#### 3.1.7. Enzymatic Degradation

Despite growing interest, the enzymatic degradation of composites and biocomposites has been little discussed in the literature and is usually focused on PLA-based biopolymers. The enzymatic degradation of bio-polymers is hypothetically an environmentally friendly end-of-life path with favourable feasibility. The recovered fibres can be recycled back into new composites, and the recovered monomers can be used for the synthesis of new polymers. Nonetheless, the efficiency of the process is quite low to achieve complete depolymerisation. Additionally, the enzymes responsible for degradation are less known, especially for synthetic resins. The TRL of this technology is estimated to be 3 for both FRP and biocomposites.

#### 3.1.8. Composting

Composting biocomposite wastes has been reported in different studies. The know-how, facilities, as well as waste volumes are already available. However, biocomposites must be composited together with other organic waste, and biocomposites are not currently accepted in composting facilities. The TRL of this technology is estimated to be 9 for FRP composites. The use of non-biodegradable or low-bio-degradable resins can be a game changer, though. The use of epoxy and phenolic resins, for instance, would lead to an estimation of 2 for the TRL of biocomposites.

#### 3.1.9. Solvolysis

Solvolysis is the most widespread chemical recycling process for polymer-based materials, as it allows for the recovery of a wide variety of valuable resins and fibres from multi-material and multi-layer plastic waste. The resin can be recovered as a monomer or oligomer, which can be used as chemical feedstock. The fibres also can be converted into new thermoplastic composites, moulding compounds, and non-woven fibre fabrics. Noting the fact that chemical recycling does not apply high mechanical and thermal forces, as in mechanical and thermal recycling, the recycled fibres have a higher length and better mechanical properties (but are not comparable with virgin fibres). On the other hand, the use of chemicals and solvents is associated with environmental burdens. This technology usually requires lower temperatures than pyrolysis to convert the polymers into monomers or other building blocks and, consequently, prevents the degradation of the retrieved fibres. Solvolysis is expected to require lower capital expenditures (CapEx) in comparison to pyrolysis and gasification. It has been investigated for both CFRP and GFRP composites and can potentially be applied to biocomposites without substantial differences by evaluating the process conditions and the required solvents. However, there is no scientific publication on the use of solvolysis for biocomposites. The TRL of this technology is estimated to be 3 or 4 for CFRP and GFRP composites and 3 for biocomposites.

#### 3.1.10. Dissolution

Dissolution can be used for multi-material and multi-layer plastic waste and allows for the recovery of both fibres and polymer resin from the composite. Nonetheless, to eliminate the surface contaminants and use the polymer for end-use applications, post-treatment processes would be needed. Dissolution shares other advantages with solvolysis, such as the environmental burdens of using solvents and low CapEx per unit product through optimum capacity design for dissolution plants that can handle a wide variety of composite and polymeric waste streams. For biofibres, the choice of solvent may be limited due to the chemical attack of the solvent on fibres. The TRL of this technology is estimated to be 3 or 4 for CFRP and GFRP composites and 2 for biocomposites.

#### 3.1.11. Comparison

**Figure 11:**
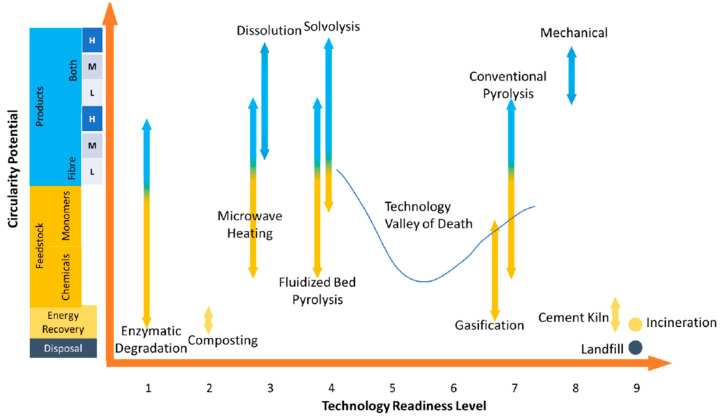
Circularity potential vs. technology readiness level (TRL) of different end-of-life paths of composites.

**Figure 12:**
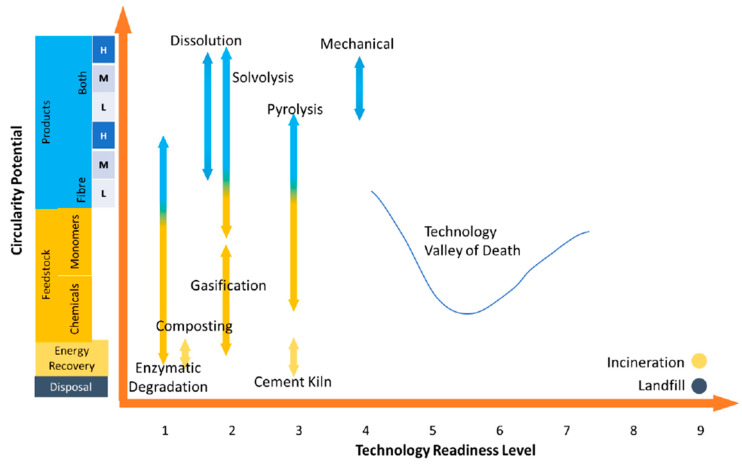
Circularity potential vs. technology readiness level (TRL) of different end-of-life paths of biocomposites.

Four of the recycling technologies, namely mechanical recycling, classic pyrolysis, gasification, and combustion in cement kilns, have high TRLs for the treatment of CFRPs and GFRPS. In the case of biocomposites, all of the technologies are still at early TRLs. On the other hand, solvolysis, dissolution, mechanical recycling, and pyrolysis (all three types) show higher circularity potential for both composites and biocomposites. Figure 11 and Figure 12 illustrate the comparison of circularity potential vs. TRL for composites and biocomposites, respectively. As can be seen in the figure, recycling technologies for biocomposites need to face and cross the “technology valley of death” to reach technological and commercial viability. This requires spending a significant amount of time on technology development, and most technologies die down as they fail to cross the “technology valley of death”.

The results of circularity and TRL assessment pointed out the importance of the market share and technical viability for recyclability in evaluating biocomposites. Moreover, the material quality and potential applications of the recycled biocomposites need to be assessed in relation to the desired specifications of the aircraft component. These points were later used in the identification of the key parameters for the MCDA and experimental testing.

### 3.2. MCDA

Table 4 shows the results of MCDA for different end-of-life paths for two biocomposites: basalt twill weave with bioepoxy resin and flax tape with bioepoxy resin. Overall, the basalt twill weave demonstrated better performance than the flax tape. This can be explained by the partial incineration of flax fibres in thermal processes, leading to fibres being lost. The technical and resource performances tended to have the highest scores for both types of composites, while the economic performance showed the lowest scores. Overall, dissolution and solvolysis showed the best performance for both evaluated biocomposites. The third alternative differed for the basalt-based (classic pyrolysis) and flax-based (mechanical recycling) biocomposites. Taking into account both biocomposites, mechanical recycling and classic pyrolysis are potential end-of-life alternatives. Energy recovery in an MSWI had the lowest performance for both composites.

### 3.3. Experimental Testing

#### 3.3.1. Laboratory Scale Testing

##### Mechanical Recycling

**Figure 13:**
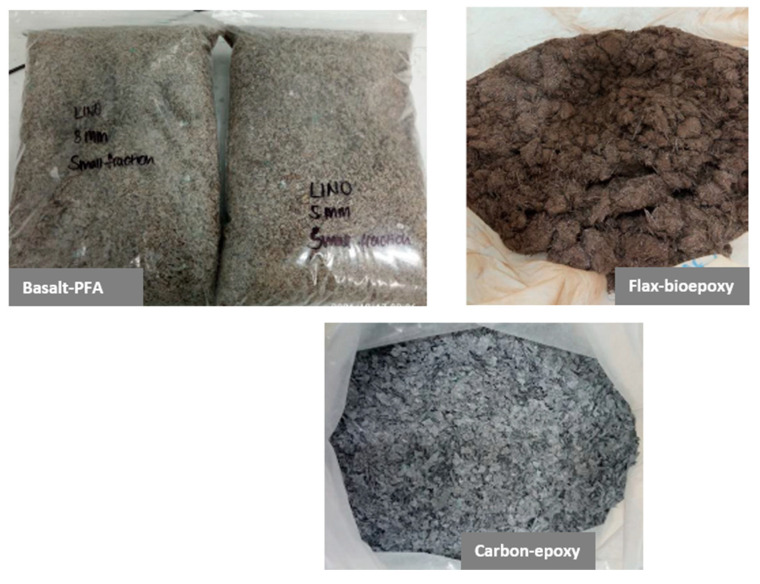
Ground biocomposites: flax bioepoxy (**top right**), basalt PFA (**top left**), carbon epoxy (**bottom**).

Figure 13 shows the ground biocomposites for three evaluated alternatives. The results of the analysis of tubes obtained from biocomposite laminates made of the ground biocomposites used as fillers show that mechanical recycling is not a suitable option for the evaluated biocomposites. The mechanical tests indicated no improvement in the mechanical properties of the biocomposite laminates, apart from the flexural properties of the flax basalt biocomposite.

##### Dissolution

**Figure 14:**
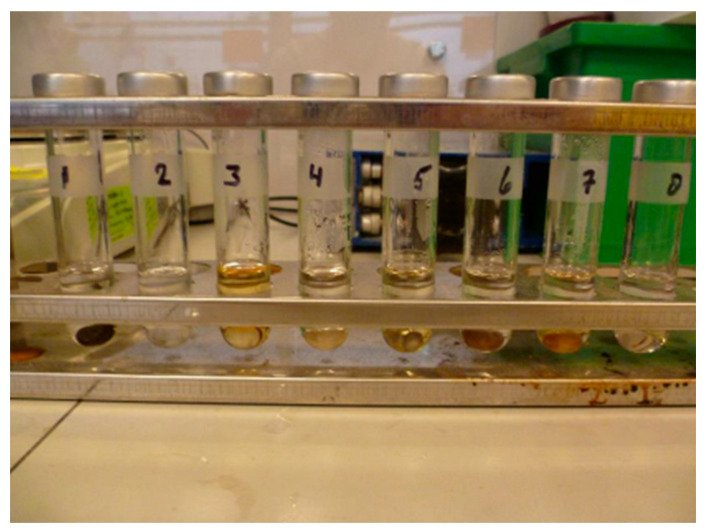
Solvent testing for the dissolution of bioepoxy resin.

**Figure 15:**
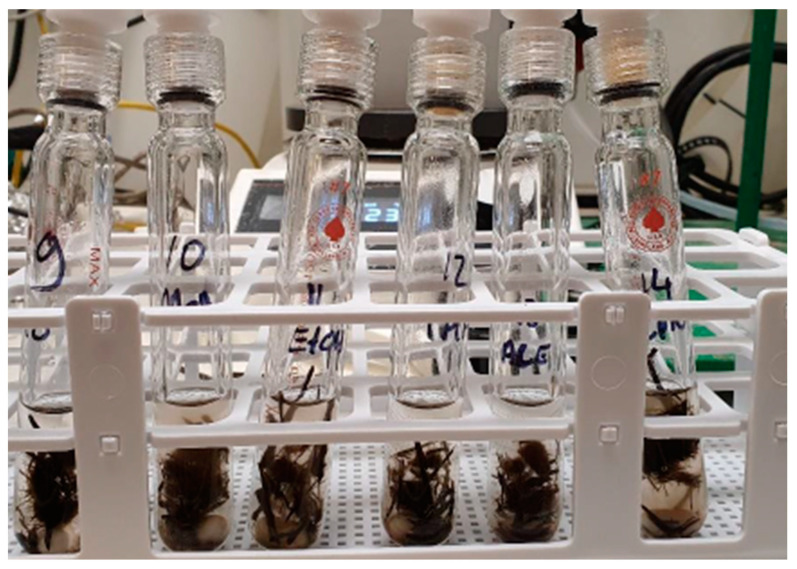
Solvent testing for the dissolution of PFA resin.

Regarding dissolution, discolouration, ranging from transparent to brown, was observed for both bioepoxy and PFA (as shown in Figure 14 and Figure 15). However, none of the tested solvents led to the dissolution of the bioepoxy resin. The resin remained intact in the test tube after the test. The results of these experiments, therefore, indicated that dissolution is not a suitable technology for the examined biocomposites and was not recommended for the pilot scale tests.

##### Solvolysis

Solvolysis testing confirmed that the evaluated biocomposites can be effectively treated at the end of their life using this method. Solvolysis showed effectiveness for all evaluated types of fibres (flax, basalt, carbon). Consequently, this technology was chosen to undergo further evaluation on a pilot scale. Similarly, the results of pyrolysis indicated the suitability of this technology for the EoL treatment of the evaluated biocomposites. Carbon and basalt fibres were retrieved in good condition, while the flax fibre demonstrated degradation and no possibility for recovery. The analysis of liquid and wax also indicated the possibility to use them as by-products in the chemical sector, such as in solvents. Therefore, solvolysis technology was selected to be evaluated further at the pilot scale.

#### 3.3.2. Pilot Scale Testing

The pilot scale results of pyrolysis showed that the fibres were retrieved in good conditions but with some residues over the fibres. This implies the need for a posterior oxidation step to eliminate these residues. The losses spotted by TGA were lower in the basalt fibre in comparison with the carbon fibre. This implies that PFA resin is easier to pyrolyse than bioepoxy. Further analysis showed that phenol is the most abundant and valuable product in the liquid fraction of pyrolysis. Similarly, the pilot scale results of solvolysis were comparable with the laboratory scale testing and showed that the fibres were retrieved in good conditions. Nonetheless, the main spotted difference was an increase in ashes and higher molecular mass in the organic residues. Comparing two processes together, the fibres after solvolysis demonstrated better conditions in comparison with pyrolysis (without an additional oxidation step).

### 3.4. Sustainability Assessment

#### 3.4.1. Life Cycle Assessment (LCA)

**Figure 16:**
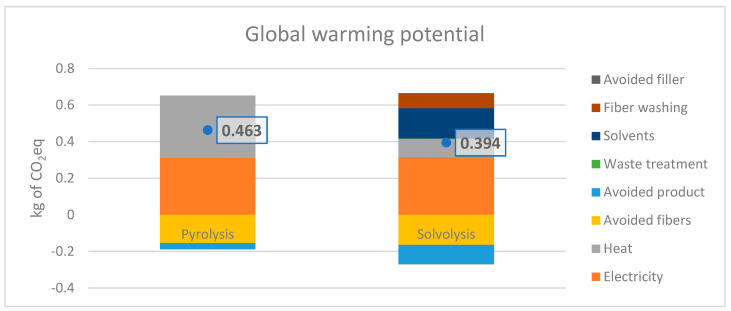
GWP of pyrolysis and solvolysis for the EoL treatment of 1 kg of basalt PFA.

The results of the GWP for pyrolysis and solvolysis are illustrated in Figure 16. Solvolysis demonstrated lower environmental impacts, i.e., 0.394 kg CO_2_eq, while pyrolysis showed 0.467 kg CO_2_eq. The difference between the two technologies was 17% and was mainly associated with the number of recovered products. While the electricity consumption was comparable (mainly due to the same pre-treatment steps), the heating energy for pyrolysis was approximately twice as much as the heat needed for solvolysis. The superiority of solvolysis can be justified by (1) a significantly lower heating demand and (2) the high rate of resin recovery, which amounts to 90% in the solvolysis process. On the other hand, the recovery rate of resin in the pyrolysis process is only around 4% (the faction turned into wax and oil), and the rest is dedicated to char. There is a high percentage of char in pyrolysis because resin contains a lot of oxygen due to its biomass origin, which lowers the generation of valuable products such as oil and wax.

#### 3.4.2. Techno-Economic Analysis (TEA)

Table 5 compares the results of TEA for both the pyrolysis and solvolysis of biocomposites. In the results, the term “alternative” refers to the size estimated based on the material and energy flows, and the term “oversized” represents the most compatible size from the model. The results indicate that both technologies are economically viable options, while the pyrolysis route has a slight advantage with a 2% lower value (the total benefit of EUR 5/kg for pyrolysis in comparison with EUR 4.91/kg for solvolysis). Moreover, there is a significantly higher cost of heating for pyrolysis, which is directly linked to a high heating demand for this technology. On the other hand, the costs associated with the capital investment are significantly higher for solvolysis, which can be justified by the high cost associated with the size of the equipment required for processing the biocomposites with solvents and washing liquids as well as solvent recovery.

## 4. Conclusions

Biocomposites are promising materials for reducing the environmental impacts of the aviation industry. Being bio-based and even biodegradable, however, does not guarantee an environmentally friendly end-of-life path. In this article, the compatibility of ten technologies for recycling biocomposite FRP waste was studied. The circularity potential and TRL assessments showed the superiority of solvolysis, pyrolysis, and mechanical recycling as the three best candidates for further evaluation. While these technologies also presented higher circularity potential, the TRLs of biocomposite recycling technologies are still not comparable when they are applied to FRP composite waste. This also points out the need for crossing the “technology valley of death” for these technologies to reach technological and commercial viability for the treatment of biocomposite waste. The quality of recycled biofibres proved to be one of the key challenging points, as they may degrade more than conventional fibres, such as carbon and glass fibres.

The results of LCA indicate the environmental benefits of solvolysis (17% less GWP) mainly due to the high recovery rate of resin as well significantly (almost half) lower heating demands in comparison with pyrolysis. Similar to LCA results, the results of TEA showed a slight advantage of solvolysis (EUR 4.91/kg) over pyrolysis (EUR 5/kg). It should be noted that, in the current business-as-usual scenarios, biocomposite waste in the aviation industry is incinerated or landfilled. Noting the fact that the European Union is intensifying landfill restrictions and plans to phase it out completely in Europe, the results of the LCA study highlight promising potentials for recovering fibres and resins toward more circularity for the aviation sector.

The results of both LCA and TEA indicate a high fraction of environmental burdens originated from pre-treatment processes, such as grinding and sieving. Future research could explore more energy efficient processes for these steps. Additionally, solvent quantity is not only crucial in the environmental burden of the solvolysis, but it also has a vital role in the sizing of the equipment and the associated costs with them. The selection of the solvent must not be limited to its effectiveness but also its downstream separation. Future research could analyse solvent separation and degradation in more detail.

## Figures and Tables

**Table 1:** The criteria for MCDA and their allocated weights per criterium and per aspect.

Aspect	Criterium	Unit	Range	Weight
Technical	Efficiency of the main product(s)	%		8.2
	Scalability of the technology	-	Very high–very low	8.1
	Quality of the main product(s)	-	Very high–very low	7.6
	Amount of hazardous waste	kg/ton		7.1
	Robustness of the operation	-	Very high–very low	6.9
	Complexity of the technology	-	Very high–very low	6.6
	Technology readiness level (TRL)	-	1 to 9	6.1
Economic	CAPEX	EUR/ton		6.5
	Recovered fibre price	EUR/ton		6.9
	Recovered resin percentage	%		7.0
	Recovered fibre percentage	%		6.9
	Revenues other products (energy, by-products)	EUR/ton		6.4
	OPEX utilities and consumables	EUR/ton		6.4
	Recovered resin price	EUR/ton		6.6
Resource	Recovered fibre percentage	%		7.6
	Recovered fibre quality	-	Very high–very low	7.0
	Additional heat consumption	MJ/ton		6.9
	Recovered resin percentage	%		7.0
	Recovered resin quality	-	Very high–very low	6.4
	External power consumption	kWh/ton		6.7
Environmental	Type of hazardous waste treatment	-	Cement kiln, hazardous waste incineration, landfill	7.1
	External power consumption	kWh/ton		6.8
	Recovered fibre percentage	%		6.9
	Recovered resin quality	-	Very high–very low	6.2
	Recovered fibre quality	-	Very high–very low	6.3
	Additional heat consumption	MJ/ton		6.4
	Recovered resin percentage	%		6.2
	Type of non-hazardous waste treatment	-	Recycling, cement kiln, incineration, landfill	5.3

**Table 2:** The allocated weights per aspect of the MCDA.

Performance	Weight
Technical	1.10
Economic	0.93
Resource	0.94
Environmental	1.02

**Table 3:** Types of solvents evaluated for dissolution.

Class of Solvent	For Both Bioepoxy and PFA	Only for PFA
Ketones	Methyl ethyl ketone	Acetone, cyclopentanone
Esters	Ethyl acetate, methyl acetate	Dimethyl carbonate, diethyl carbonate,
Sulphur compounds	Dimethyl sulfoxide	
Nitrogen compounds	N-methyl-2-pyrrolidone, dimethylformamide	
Aromatic hydrocarbons	Xylene	Toluene
Ethers	THF	
Alcohols		Methanol, ethanol, 1-propanol

**Table 4:** MCDA results of different end-of-life paths for basalt fabric and flax tape bioepoxy composites, and the average overall score for these two alternatives. Dark green indicates the best score and yellow the least score. The two highest scores per alternative are indicated by an orange border.

Composite	EoL Technology	Cement Kiln	Dissolution	Energy Recovery (MSWI)	Enzymatic Degradation	Gasification	Mechanical Recycling	Pyrolysis, Classic	Pyrolysis, Fluidised Bed	Pyrolysis, Microwave	Solvolysis	Average
Basalt twill weave—bio-epoxy	Technical	2.5	2.1	2.3	1.6	2.4	2.3	2.2	1.8	1.6	2.2	2.1
	Economic	1.2	2.2	0.9	0.9	1.3	0.7	1.6	1.5	1.7	1.9	1.4
	Resource	1.6	3	1.6	2.2	2.3	2.8	2.4	2.6	2.6	2.6	2.4
	Environmental	1.5	2.7	1.7	2	2	2.3	2	2	2.1	2.4	2.1
	Overall	1.7	2.5	1.6	1.7	2	2	2.1	2	2	2.3	2.0
Flax tape—bio-epoxy	Technical	2.1	2.2	2.3	1.3	2.1	2.3	2	1.3	1.1	2.2	1.9
	Economic	1.2	2.2	0.9	1	0.9	0.7	1.3	1.5	1.2	1.9	1.3
	Resource	1.6	3	1.6	2.2	1.3	2.8	1.4	1.5	1.5	2.4	1.9
	Environmental	1.5	2.7	1.7	2	1.6	2.3	1.4	1.4	1.4	2.3	1.8
	Overall	1.6	2.5	1.6	1.6	1.5	2	1.5	1.4	1.3	2.2	1.7
Average score		1.7	2.5	1.6	1.7	1.8	2.0	1.8	1.7	1.7	2.3	1.9

**Table 5:** The comparison of the TEA of pyrolysis and solvolysis.

Parameter	Unit	Pyrolysis	Solvolysis
Heating	MJ/kg feedstock	6.450	1.910
Electricity	kWh/kg feedstock	1.092	1.115
Energy costs	EUR/kg feedstock	0.090	0.066
Capital Investment	Million euros	2.726 (alternative) 5.235 (oversized)	8.021
Total benefit	EUR/kg feedstock	5.00	4.910
Production costs	EUR/kg feedstock	0.292 (oversized) 0.256 (alternative)	0.792
Net benefits	EUR/kg feedstock	4.71 (oversized) 4.74 (alternative)	4.12

## Appendix A

**Table A1:** Inventory data for the pyrolysis process.

Input Element	Value	Unit	Ecoinvent Dataset	Source
Energy consumption for grinding	0.94	kWh/kg input	Electricity, medium voltage, Europe (Eurostat)	Primary data
Energy consumption for sieving	0.15	kWh/kg input	Electricity, medium voltage, Europe (Eurostat)	Secondary data [17]
Energy consumption for pyrolysis	6.64	MJ/kg input	Heat, district or industrial, natural gas {RER}|market group for|APOS, U	Primary data
Recovered basalt fibres	0.83	kg/kg input	Basalt fibres	Primary data
Avoided product-pyrolysis oil	0.008	kg/kg input	Heavy fuel oil {Europe without Switzerland}|	Primary data
Avoided product—Wax	0.03	kg/kg input	Petroleum slack wax {Europe without Switzerland}|	Primary data
Avoided product—fillers	0.02	kg/kg input	Lime {Europe without Switzerland}|lime production, milled, loose	Primary data
CO_2_ emissions, biogenic	0.19	kg/kg input		Calculated

**Table A2:** Inventory data for the solvolysis process.

Input Element	Value	Unit	Ecoinvent Dataset	Source
Energy consumption for grinding	0.94	kWh/kg input	Electricity, medium voltage, Europe (Eurostat)	Primary data
Energy consumption for sieving	0.15	kWh/kg input	Electricity, medium voltage, Europe (Eurostat)	Secondary data [17]
Solvolysis heat consumption	0.53	kWh/kg input	Heat, district or industrial, natural gas {RER}	Primary data
Glacial acetic acid solvent	8.03	kg/kg input	Acetic acid, without water, in 98% solution state {GLO}	Primary data
Hydrogen peroxide solvent	1.02	kg/kg input	Hydrogen peroxide, without water, in 50% solution state {RER}	Primary data
Water (additive to solvolysis)	4.15	kg/kg input	Water, deionised {Europe without Switzerland}	Primary data
Acetone for washing fibres	1.84	kg/kg input	Acetone, liquid {RER}|market for acetone, liquid	Primary data
Recovered fibres	0.84	kg/kg input	Basalt fibres	Primary data
Recovered acetic acid	8.00	kg/kg input	Acetic acid, without water, in 98% solution state {GLO}	Primary data
Recovered water	4.15	kg/kg input	Water, deionised {Europe without Switzerland}	Primary data
Recovered hydrogen peroxide	0.92	kg/kg input	Hydrogen peroxide, without water, in 50% solution state {RER}	Primary data
Recovered acetone	1.81	kg/kg input	Acetone, liquid {RER}|market for acetone, liquid	Primary data
Waste water treatment of losses	0.12	kg/kg input	Wastewater, average {Europe without Switzerland}	Secondary data (Ecoinvent)
Incineration of fibre loss	0.05	kg/kg input	Waste glass {CH}|treatment of, municipal incineration	Secondary data (Ecoinvent)
Recovery of residue	0.09	kg/kg input	Petroleum slack wax {Europe without Switzerland}|	Primary data
Avoided product—fillers	0.02	kg/kg input	Lime {Europe without Switzerland}|lime production, milled, loose	Primary data
